# Association between Sarcopenia and Poor Glycemic Control in Older Adults with Type 2 Diabetes Mellitus

**DOI:** 10.3390/diseases11040175

**Published:** 2023-11-30

**Authors:** Fabián Alonso Alfaro-Alvarado, José Vicente Rosas-Barrientos, María Esther Ocharan-Hernández, Dylan Díaz-Chiguer, Cruz Vargas-De-León

**Affiliations:** 1 Laboratorio de Modelación Bioestadística para la Salud, Sección de Estudios de Posgrado, Escuela Superior de Medicina, Instituto Politécnico Nacional, Ciudad de México 11340, Mexico; drgeriatric.fa@gmail.com (F.A.A.-A.); estherocharan@hotmail.com (M.E.O.-H.); 2 Clínica Hospital No. 24, Instituto de Seguridad y Servicios Sociales de los Trabajadores del Estado, Ciudad Guzmán 49097, Mexico; 3 Hospital Regional 1° de Octubre, Instituto de Seguridad y Servicios Sociales para los Trabajadores del Estado, Ciudad de México 07760, Mexico; diploestadis@gmail.com; 4 Dirección Normativa de Salud, Instituto de Seguridad y Servicios Sociales para los Trabajadores del Estado, Ciudad de México 06030, Mexico; dra.dylandiaz@outlook.com; 5 División de Investigación, Hospital Juárez de México, Ciudad de México 07760, Mexico

**Keywords:** sarcopenia, poor glycemic control, handgrip strength, muscle mass, type 2 diabetes

## Abstract

Background: Aging is associated with a decrease in muscle mass. Insulin resistance and hyperglycemia accelerate muscle loss, leading to a deterioration in strength, muscle mass, and physical capacity in older adults. This study was conducted to determine the association between sarcopenia and poor glycemic control in older adults with type 2 diabetes mellitus (T2D). Methods: A cross-sectional study was carried out in older adults with T2D in geriatric outpatient clinics. Sarcopenia was diagnosed as per the European Working Group on Sarcopenia in Older People 2 (EWGSOP2) criteria. According to glycosylated hemoglobin (HbA1c) levels, participants were classified into glycemic control (HbA1c ≤ 7.5%) and poor glycemic control (HbA1c ≥ 7.5%) groups. Results: Older adults with sarcopenia were found to have poor glycemic control compared to adults without sarcopenia (62.3% vs. 47.9%, *p* = 0.007). Logistic regression analysis showed an association between poor glycemic control and the presence of sarcopenia (odds ratio (OR): 1.79, 95% confidence interval (CI): 1.17–2.75) and low muscle mass (OR: 1.73, 95% CI: 1.07–2.73). Conclusions: Poor glycemic control is associated with the presence of sarcopenia and low muscle mass, which highlights the need to implement better treatment strategies in order to reduce the loss of muscle mass.

## 1. Introduction

Sarcopenia is defined as the loss of muscle mass and strength related to the aging process [1]. The worldwide prevalence rate of sarcopenia is estimated to be 10% [2]; in Mexico, it is estimated that 33.6% of the population, mainly women, suffer from it, increasing from the age of 80 years old [3]. It is accompanied by a high burden of comorbidity, with cardiovascular disease, T2D, and neurodegenerative diseases being the most frequent conditions [4,5].

Aging is related to a decrease in glucose tolerance, as well as factors such as an increase in adiposity, particularly in the abdominal region, accompanied by a decrease in muscle mass, causing a deterioration in glucose regulation [6]. It has been determined that between 30% and 40% of a person’s muscle mass can decrease by the time they reach an age of 80 years. It is reasonable to think that this loss is related to the development of glucose intolerance and subsequently to the increased risk of developing T2D [7]. Insulin resistance is associated with an increased loss of appendicular lean mass in both men and women [8,9,10]. People with prediabetes and high HbA1c levels experience a loss of muscle mass, muscle strength, and physical performance, particularly in the lower extremities among older ages, and worsening when untreated [11,12,13,14,15].

Sarcopenia is more frequent in patients with diabetes than in normoglycemic patients. Both type 1 diabetes mellitus (T1D) and T2D patients show lower handgrip strength and muscle mass [16], suggesting an inverse correlation between appendicular muscle mass with diabetes duration and fat mass, as well as a positive correlation with appendicular muscle mass based on body mass index (BMI), physical activity level, and muscle strength [17], with T2D being a risk factor for developing sarcopenia (37%) and pre-sarcopenia (73%) compared to individuals without T2D [18]. Other factors, such as female gender [19,20], T1D [16,17], age > 65 years old [18,19], a high BMI [17,21,22,23], hypoalbuminemia [24,25], poor nutritional status [19,20,21,22,23,24], low levels of physical activity [19,22,24,25,26], and high insulin requirements [27], are risk factors for the development of sarcopenia, which was independently associated with short-term mortality after hospital discharge [28].

A progressive increase in HbA1c levels was inversely associated with a low percentage of total, appendicular, and trunk lean muscle mass [13,29]. The same findings were observed in subjects with no previous diagnosis of T2D but who had hyperglycemia and T2D without seeking treatment [29]. In addition, low muscle mass, low handgrip strength, and insulin resistance were independent factors determining poor glycemic control [30] and higher glucose fluctuations [31]. A >1% decrease in HbA1c levels was found to improve muscle mass and gait speed [32]. Conversely, patients with microangiopathic complications have a significantly increased risk of sarcopenia, especially when diabetic retinopathy, nephropathy, and peripheral neuropathy are present [12,24,33].

Insulin use has been shown to be an independent factor in decreasing muscle mass [27,32]. Lower handgrip strength and gait speed were observed when patients were treated with insulin [25]. However, insulin sensitizers and dipeptidyl-peptidase-4 inhibitors (iDPP-4s) minimize the loss of strength and muscle mass [26,34,35]. No association has been found between the duration of T2D and the development of sarcopenia in those within the age range of 6 to 15 years [16] or with the use of other oral antidiabetics [36]. This study was conducted to determine the association between sarcopenia and poor glycemic control in older adults with T2D.

## 2. Materials and Methods

### 2.1. Study Participants

A cross-sectional study was conducted in older adults with T2D, recruited from the Geriatrics outpatient clinic of Hospital 24 del Instituto de Seguridad y Servicios Sociales de los Trabajadores del Estado (ISSSTE, for its acronym in Spanish), Ciudad Guzmán, Mexico. The participants were 60 years old or older with a confirmed diagnosis of T2D, consecutively assessed from July 2022 to June 2023. The inclusion criteria were as follows: (1) an age of ≥60 years and (2) a diagnosis of T2D (according to the American Diabetes Association criteria) [37]. The exclusion criteria were as follows: (1) the presence of severe physical or cognitive limitations, (2) the presence of an acute process that warrants emergency care or hospital admission, (3) the diagnosis of terminal illness or being in palliative care, and (4) an inconclusive diagnosis of T2D or the absence of antidiabetic treatment at the time of assessment.

Our study procedures were approved by our institution’s Ethics and Research Committees under the registration number DJSMEI-13149. All participants agreed and signed a written informed consent form.

### 2.2. Clinical Features

Each participant was questioned directly or through the main caregiver to acquire demographic variables, as well as their age, medical history, and medication records. Comorbidity was defined through the Charlson Comorbidity Index as ≥3 diseases [38] and the presence of polypharmacy, reporting the consumption of ≥5 medications simultaneously in the last month. The duration of T2D was dichotomously defined as <20 years and >20 years. According to the medical record, the presence of complications related to T2D was recorded: retinopathy, neuropathy, cardiopathy (heart failure, myocardial infarction, angina pectoris, atrial fibrillation); cerebrovascular complications (transient ischemia, cerebrovascular event); angiopathy (peripheral arterial disease in upper or lower limbs, carotid stenosis); nephropathy (estimated GFR via CKD-EPI of creatinine ≤ 60 mL/min/1.73 m^2^ without dialysis). Weight and height were measured, and BMI was calculated as weight (kg)/height^2^ (m). The nutritional status of older adults was assessed through the Mini Nutrition Assessment (MNA); a score of ≥18 was defined as adequate nutritional status, and a score of ≤17 was defined as malnutrition [39]. Frailty was defined through the FRAIL (Fatigue, Resistance, Ambulation, Illness, and Loss of weight) scale; a score of ≥3 points was categorized as frailty [40]. Upon direct questioning at the time of care, physical activity was assessed by calculating metabolic equivalents (METs) through the Duke Activity Status Index (DASI). The calculation was as follows: METs = total DASI score × 0.43 + 9.6/3.5, low physical activity was categorized if the METs were ≤5 [41].

The biochemical parameters of interest, such as HbA1c, total cholesterol, high-density cholesterol (HDL-c), low-density cholesterol (LDL-c), triglycerides, uric acid, and albumin, were obtained from the clinical record less than 3 months from the date they were taken. Insulin resistance was measured through the triglyceride/glucose (TyG) index, according to the following formula: Ln (TG [mg/dL] x glucose [mg/dL]/2), with a value of ≥8.80 defined as insulin resistance [42]. Glycemic control was determined through HbA1c levels ≤ 7.5%, and poor glycemic control as an HbA1c ≥ 7.5%. 

### 2.3. Definition of Sarcopenia

According to the EWGSOP2 criteria [1], older adults with low muscle strength and low muscle mass were defined as having sarcopenia. Muscle strength was defined through the handgrip strength of the dominant hand, using a JAMAR^®^ dynamometer. A maximum of 3 attempts were performed, recording the highest value for our subsequent analyses. The cut-off value for low muscle strength was ≤16 kg for women and ≤27 kg for men. The appendicular skeletal muscle mass (ASM) was determined through the formula ASM kg = 0.215 × calf circumference (cm) + 0.093 × handgrip strength (kg) + 0.061 × weight (kg) + 3.637 × sex + 0.112 × height (cm) − 16.449; where sex: male = 1; female = 0. We defined low muscle mass as men with ≤20 kg and women with ≤15 kg [43].

Physical performance was measured by using walking speed in meters/second (m/s), timed over four linear meters. Each older adult was instructed and assessed on 2 occasions; the best-timed record was used to define poor physical performance. The cutoff value was set at ≤0.8 m/s.

### 2.4. Statistical Analysis

The Kolmogorov–Smirnov test was performed to assess the distribution of the variables. The calculated values were presented as frequencies and percentages or means and standard deviations (SDs). Student’s *t*-test and Chi-square were used for the comparison of numerical and categorical variables, respectively. The analysis of association was performed through logistic regression models with response variables: sarcopenia and its components. The odds ratios (OR) and 95% confidence intervals (CI) were estimated as a measure of the effect size of the poor glycemic control in the logistic regression. We adjusted the estimates of the OR of poor glycemic control using age, sex, and variables related to T2D. We performed a sensitivity analysis of the estimate of the OR of poor glycemic control adjusting to a different variable related to T2D. The Hosmer–Lemeshow test was used to assess the goodness of fit of the logistic regression models. All statistical analyses were performed by using the IBM SPSS Statistics 29.0.1 software version. Any *p*-value < 0.05 was considered significant.

## 3. Results

### 3.1. Characteristics of the Participants

**Figure 1:**
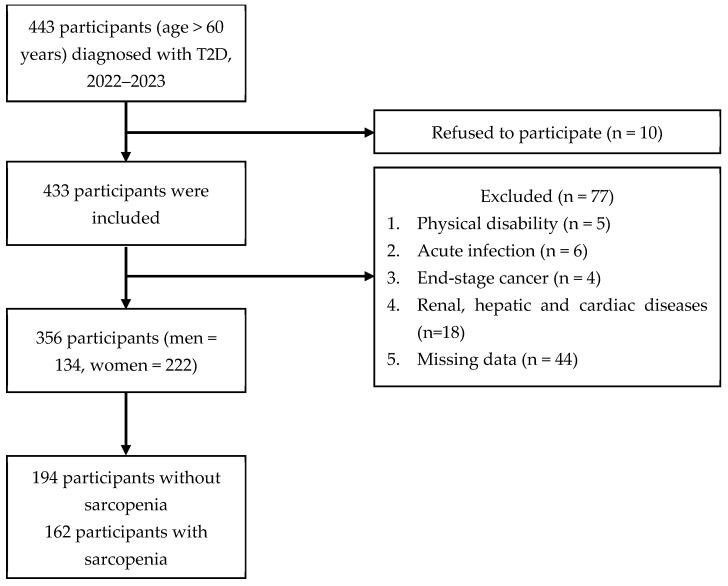
Flow diagram for study participants.

Figure 1 shows that 443 patients diagnosed with T2D were initially recruited for the study, of whom 87 were eliminated and 356 participants were enrolled.

The general characteristics of the patients according to the diagnosis of sarcopenia are presented in Table 1. The frequency of sarcopenia in our study was 45.5%, of which 97 cases were women (59.9%) and 65 cases were men (40.1%). The overall poor glycemic control in patients was 54.5%.

When comparing the groups with or without sarcopenia, it was observed that patients with the presence of sarcopenia were older (*p* < 0.001), had poor glycemic control (62.3% vs. 47.9%, *p* = 0.007), burden of comorbidity (*p* = 0.014), malnutrition (*p* < 0.001), and frailty (*p* < 0.001), as well as a longer duration of T2D (*p* < 0.001). They also presented a higher frequency of complications associated with T2D in general (81.5% vs. 64.4%, *p* < 0.001), of which heart disease (26.5% vs. 11.9%, *p* < 0.001) and nephropathy (21.0% vs. 12.9%, *p* < 0.001) were the most frequent. Older adults with sarcopenia registered lower physical activity measured through METs (4.2 vs. 5.6, *p* < 0.001). 

Conversely, body composition in sarcopenia patients revealed that BMI (*p* < 0.001), waist circumference (*p* < 0.001), hip circumference (*p* < 0.001), and body fat percentage (*p* < 0.001) were lower. For sarcopenia-defining components, the means for handgrip strength (13.9 kg vs. 23.4 kg, *p* < 0.001), appendicular skeletal muscle mass (14.2 kg vs. 16.9 kg, *p* < 0.001), and gait speed (0.60 m/s vs. 0.82 m/s, *p* < 0.001) were lower than in those older adults without sarcopenia. 

HbA1c levels were higher in patients with sarcopenia (8.2% vs. 7.8%, *p* = 0.027). However, their albumin, total cholesterol, and triglyceride levels were lower than those without sarcopenia. The use of antidiabetic drugs showed that the frequency of insulin use was higher in patients with sarcopenia (68.5% vs. 44.3%, *p* < 0.001) and a lower use of sulfonylureas and biguanides.

### 3.2. Components of Sarcopenia According to Glycemic Control

We compared the components that define the diagnosis of sarcopenia with glycemic control. In patients with poor glycemic control, handgrip strength (13.5 kg vs. 23.5 kg, *p* < 0.001), appendicular skeletal muscle mass (14.0 kg vs. 16.9 kg, *p* < 0.001), and gait speed (0.62 m/s vs. 0.80 m/s, *p* < 0.001) were significantly lower for subjects with sarcopenia. In patients with glycemic control, there were no significant differences between subjects with and without sarcopenia for handgrip strength (13.5 kg vs. 14.7 kg, *p* = 0.091), for appendicular skeletal muscle mass (14.0 kg vs. 14.5 kg, *p* = 0.486), and for gait speed (0.62 m/s vs. 0.63 m/s, *p* = 0.701).

### 3.3. Association of Sarcopenia Risk and Its Components with Glycemic Control

Logistic regression analysis for sarcopenia and its components focused on poor glycemic control (HbA1c ≥ 7.5%), the associations are shown in Table 2. Table A1 of the Appendix A shows the results of the Hosmer–Lemeshow test. Poor glycemic control was significantly associated with the presence of sarcopenia and with low muscle mass, without being significant for low muscle strength and low gait speed. The same associations held for both sarcopenia and low muscle mass, when adjusted for age and sex, and with variables such as comorbidity, T2D-related complication, presence of heart disease, nephropathy, and duration of T2D ≥ 20 years old. 

## 4. Discussion

In our study, a high frequency of sarcopenia was observed in older adults with T2D, having a significant association with some clinical characteristics such as advanced age, higher comorbidity burden, malnutrition status, presence of frailty, low physical activity, presence of complications associated with T2D, and a disease duration of more than 20 years. More than half of the patients presented poor glycemic control, represented by an HbA1c level ≥ 7.5%, this being more frequent in those older adults who were categorized with sarcopenia. 

Poor glycemic control was significantly associated with sarcopenia and low muscle mass (OR 1.79 and OR 1.73, respectively), but not with low muscle strength or low gait speed; these associations were maintained when adjusted for variables related to T2D itself. 

The frequency of sarcopenia in our study was 45.5%, which is higher than the 11% reported worldwide [2] and 33.6% in our country [3]. This variability in prevalence arises from the heterogeneity of the criteria used to define sarcopenia. Increasing age and female sex are the non-modifiable factors most frequently associated with sarcopenia found in our study and are similarly referred to in other cross-sectional studies [18,19,20]. In Mexico, Perez-Zepeda et al. found that the prevalence of sarcopenia increases with age, with increasing values between 60 and 69 years old (16.06%), 70 and 79 years old (32.85%), and over 80 years old (51.01%) [44]. Body composition and muscle mass vary according to cultural, regional, and geographical location in our country, both for men and women, and muscle mass was higher for adults living in the center of the country [45]. 

In the present study, the presence of sarcopenia was further associated with a higher burden of comorbidity (Charlson index ≥ 3 diseases), malnutrition status (MNA ≤ 17 points), presence of frailty (FRAIL ≥ 3 points), clinical conditions related to the natural history of T2D (prolonged duration of the disease, presence of chronic complications), and low physical activity. These same findings have been demonstrated in several studies, where, when evaluating subjects with T2D, the risk of developing sarcopenia was OR 1.55; 95% CI 1.25–1.91, *p* < 0.001; it is noteworthy that subjects with T2D presented lower physical performance and muscle strength than those with euglycemia, without observing a difference in the amount of muscle mass [25].

Prolonged periods of sedentary lifestyle, poor nutritional status, and lower levels of physical activity have been identified as common risk factors for the presence of sarcopenia, which is exacerbated in the presence of T2D [19]. In a study with similar findings, subjects with sarcopenia have a higher risk of malnutrition than those with normal nutritional status; similarly, in the subgroup of women with malnutrition (OR 4.97; *p* = 0.003) and women with T2D (OR 5.52; *p* = 0.019), they were more likely to have sarcopenia [20]. Nutritional status is perhaps the most important determinant associated with sarcopenia. In our study, older adults with T2D who presented sarcopenia were associated with a state of malnutrition; this association is dependent on BMI, since obesity has bimodal behavior, it increases the risk of presenting sarcopenia (OR 3.2; 95% CI 1.24–8.26) [21]. However, high BMI also had a negative association with the development of sarcopenia (men, OR 0.57, 95% CI 0.44–0.73; women, OR 0.48, 95% CI 0.33–0.70) [46]; in addition, lower BMIs, are associated with the development of sarcopenia [22], as nutritional status itself has shown a positive correlation with muscle mass and handgrip strength [20,23,24]; the same relationship holds with low protein intake and lower physical activity [20,47,48]. We defined low physical activity as ≤5 METs by the Duke index, with the mean being lower in subjects with sarcopenia; however, in the multivariate analysis, it was not associated with the presence of sarcopenia, low muscle strength, low muscle mass, or low gait speed. Nevertheless, several studies have reported that a state of pre-frailty (OR 4.75; 95% CI 1.90–11.89; *p* = 0.001) [49] and low physical activity (OR 15.35; 95% CI 1.69–139.47; *p* = 0.015) increases the risk of sarcopenia [19,22,24,25,26].

Insulin resistance determines the onset of accelerated skeletal muscle loss [8,9,10]. This loss is more pronounced in the appendicular area, especially in the lower extremities, and this deterioration is substantially more aggressive in patients without antidiabetic treatment. Park SW et al., in their study, showed in a 6-year follow-up that loss of total muscle mass was more pronounced in older adults with T2D but without treatment than in those with T2D receiving treatment and those without T2D (−435 ± 79 vs. −293 ± 72 vs. −193 ± 22 gr/year, respectively, *p* < 0.01) [13]. In our study, insulin resistance was evaluated via the TyG index; the mean values found for both groups determine the presence of insulin resistance. However, it was slightly higher in patients without sarcopenia (9.1 vs. 9.3; *p* = 0.046). Another observation was made for body fat index, where patients with sarcopenia registered a lower percentage for calculated body fat (38.4% vs. 42.2%, *p* < 0. 001), a finding that contrasts with what was found in a recent study, which evaluated the association between body fat percentage and sarcopenia; logistic regression analyses demonstrated that a high body fat percentage was associated with an increased risk of sarcopenia for both sexes (male, OR 1.38, 95% CI 1.15–1.65; female, OR 1.30, 95% CI: 1.07–1.56) [46].

In our study, poor glycemic control was associated with the presence of sarcopenia and low muscle mass among patients. Studies have highlighted the importance of quantitative muscle importance with the risk of developing T2D. Son JW et al. demonstrated in a follow-up of more than 9 years in middle-aged adults without T2D that the presence of low muscle mass increases the risk of developing T2D by 11.9% in non-obese patients and 19.7% in patients with obesity [50]. Cross-sectional studies have also shown this association [13,14,15], as well as an association with low muscle strength [51]. Persistent hyperglycemia and poor glycemic control are determinants for the development of sarcopenia in the elderly; several studies show that higher glucose and HbA1c levels are associated with poorer muscle quality in quantitative aspects, muscle strength, and physical performance [15,16,25,29,32,33]. Low muscle strength was associated with a higher total insulin dose requirement [30], and low muscle mass was associated with fluctuations in glucose levels and greater variability in fasting glucose ranges [31].

Several studies have shown that the class of drugs including iDPP-4 has a neutral and/or attenuating effect on the loss of muscle mass [34,35]; however, in the case of iSGLT-2, a reduction in muscle mass has been reported [52]. The effect of insulin treatment remains controversial; in cross-sectional designs, the use of insulin shows an association with the development of sarcopenia [32]. Nonetheless, in longitudinal designs, insulin treatment and reduction of HbA1c levels have been shown to attenuate the progression of sarcopenia in older adults with T2D [53,54].

Our study was conducted in an older Mexican population and sought to determine the association between sarcopenia and T2D in a context that may differ from the settings of many previous studies. Despite the existing body of evidence, verifying the reproducibility of findings in various populations is paramount to ensure the external validity of research, providing a stronger foundation for translating findings into clinical practice. This is crucial for clinicians and policymakers seeking evidence applicable to diverse demographic and ethnic groups.

Our study has the following limitations: the number of patients assessed is relatively small in terms of giving external validity to the findings in older Mexican adults with T2D. Due to the type of study, only glycemic control was determined at the time of assessment; a longitudinal study could be necessary to assess changes in HbA1c levels, time of glycemic control, adherence to treatment, and the combinations of the different antidiabetic drugs and their doses. As insulin was found to be a deleterious factor in muscle mass and sarcopenia, it was necessary to determine the influence of the type of insulin used, the doses, and the scheme employed. In determining malnutrition as an independent implication of glycemic control, it was necessary to describe the caloric quantity and protein intake per day since it directly influences glucose control and the development of sarcopenia. In contrast, we evaluated physical activity through the estimation of METs performed for certain activities of daily living. However, we did not evaluate the intensity, frequency, and duration nor the history of physical exercise since the level of physical activity can modify changes in muscle mass and strength. Finally, our study did not use any sophisticated method in determining body composition (dual-energy X-ray absorptiometry, bioelectrical impedance) or other imaging methods.

## 5. Conclusions

In summary, the frequency of poor glycemic control in older adults with T2D was higher when they presented sarcopenia and low muscle mass. Our findings indicate that poor glycemic control is associated with sarcopenia and low muscle mass, which determines the need to implement better treatment strategies to reduce the loss of muscle mass.

## Figures and Tables

**Table 1:** Clinical characteristics of older adults with or without sarcopenia.

Feature	Sarcopenia (*n* = 162)	No Sarcopenia (*n* = 194)	*p*-Value ^†^
Age (years)	77.8 (7.2)	72.6 (7.8)	<0.001
Women (%)	97 (59.9)	125 (64.4)	0.377
Comorbidity (Charlson ≥ 3)	129 (79.6)	132 (68.0)	0.014
Malnutrition (MNA ≤ 17)	121 (74.7)	89 (45.9)	<0.001
Fragility (FRAIL ≥ 3)	90 (55.6)	38 (19.6)	<0.001
Polypharmacy (≥5 drugs)	121 (74.7)	141 (72.7)	0.668
Duration of T2D (years)	18.2 (9.8)	14.2 (9.1)	<0.001
Physical Activity (METs)	4.2 (0.9)	5.6 (1.4)	0.001
TyG Index	9.1 (0.5)	9.3 (0.6)	0.046
Poor glycemic control (HbA1c ≥ 7.5%)	101 (62.3)	93 (47.9)	0.007
T2D-related complication (%)	132 (81.5)	125 (64.4)	<0.001
Retinopathy (%)	24 (14.8)	20 (10.3)	0.198
Neuropathy (%)	85 (52.5)	92 (47.4)	0.343
Heart disease (%)	43 (26.5)	23 (11.9)	<0.001
Cerebrovascular (%)	28 (17.3)	24 (12.4)	0.191
Angiopathy (%)	34 (21.0)	32 (16.5)	0.277
Nephropathy (%)			0.138
>60 mL/min/1.73 m^2^	128 (79.0)	169 (87.1)	
45–59 mL/min/1.73 m^2^	3 (1.9)	1 (0.5)	
30–44 mL/min/1.73 m^2^	18 (11.1)	11 (5.7)	
16–29 mL/min/1.73 m^2^	13 (8.0)	13 (6.7)	
<15 mL/min/1.73 m^2^	0 (0)	0 (0)	
Anthropometry			
BMI (kg/m^2^)	25.2 (4.7)	28.9 (5.4)	<0.001
Waist circumference (cm)	94.2 (11.8)	102.6 (13.5)	<0.001
Hip circumference (cm)	101.5 (12.2)	108.4 (10.9)	<0.001
Body Fat (%)	38.4 (8.6)	42.2 (8,4)	<0.001
Handgrip strength (kg)	13.9 (4.3)	23.4 (6.8)	<0.001
ASM (kg)	14.2 (3.3)	16.9 (3.9)	<0.001
Gait speed (m/s)	0.60 (0.2)	0.82 (0.3)	<0.001
Biochemical markers			
HbA1c (%)	8.2 (1.8)	7.8 (1.8)	0.027
Albumin (g/dL)	3.7 (0.4)	3.9 (0.4)	<0.001
Cholesterol (mg/dL)	157.9 (42.4)	169.8 (43.5)	0.010
HDL-c (mg/dL)	43.5 (11.9)	44.1 (10.8)	0.589
LDL-c (mg/dL)	85.0 (33.9)	91.0 (33.6)	0.095
Triglycerides (mg/dL)	147.3 (54.8)	172.9 (80.6)	<0.001
Uric acid (mg/dL)	6.0 (1.9)	5.8 (1.7)	0.340
Antidiabetic medication			
Sulfonylureas (%)	1 (0.6)	11 (5.7)	0.009
Biguanides (%)	115 (71.0)	157 (80.9)	0.028
Thiazolidinediones (%)	1 (0.6)	3 (1.5)	0.408
DPP-4 inhibitors (%)	112 (69.1)	128 (66.0)	0.527
SGLT2 inhibitors (%)	59 (36.4)	59 (30.4)	0.231
GLP-1 analogs (%)	5 (3.1)	2 (1.0)	0.164
Insulin (%)	111 (68.5)	86 (44.3)	<0.001

Abbreviations: ASM: appendicular skeletal muscle mass; BMI: body mass index; T2D: diabetes mellitus 2; GLP-1: glucagon-like peptide receptor type 1 agonists; HbA1c: glycosylated hemoglobin; HDL-c: high-density cholesterol; iDPP4: dipeptidyl-peptidase 4 inhibitors; iSGLT2: sodium-glucose cotransporter type 2 inhibitors; LDL-c: low-density cholesterol; METs: metabolic equivalent; MNA: Mini-Nutritional Assessment; TyG: glucose/triglyceride index. Results are expressed as frequency (%) or mean (standard deviation, SD). † *p* value (t Student or Chi-square test).

**Table 2:** Association between poor glycemic control and sarcopenia and its components.

	Sarcopenia		Low Muscle Strength		Low Muscle Mass		Low Gait Speed	
	OR (95% CI)	*p*-Value	OR (95% CI)	*p*-Value	OR (95% CI)	*p*-Value	OR (95% CI)	*p*-Value
PC	1.79 (1.17–2.75)	0.007	1.44 (0.94–2.19)	0.088	1.73 (1.07–2.73)	0.016	1.24 (0.81–1.89)	0.315
PC, age, sex	1.79 (1.14–2.81)	0.011	1.45 (0.92–2.28)	0.107	1.73 (1.06–2.82)	0.027	1.18 (0.76–1.83)	0.462
PC, age, sex, comorbidity	1.80 (1.14–2.83)	0.011	1.45 (0.91–2.30)	0.111	1.74 (1.06–2.83)	0.026	1.16 (0.74–1.81)	0.514
PC, age, sex, malnutrition	1.48 (0.93–2.37)	0.098	1.14 (0.71–1.85)	0.574	1.53 (0.92–2.53)	0.095	0.99 (0.62–1.57)	0.973
PC, age, sex, frailty	1.54 (0.96–2.47)	0.073	1.11 (0.67–1.84)	0.659	1.56 (0.95–2.57)	0.077	0.91 (0.56–1.48)	0.731
PC, age, sex, DM complication	1.63 (1.03–2.58)	0.037	1.32 (0.31–2.10)	0.239	1.54 (0.93–2.54)	0.090	1.10 (0.70–1.73)	0.657
PC, age, sex, activity ≤ 5 METs	1.57 (0.96–2.56)	0.067	1.20 (0.73–1.98)	0.457	1.48 (0.89–2.48)	0.128	0.98 (0.61–1.58)	0.962
PC, age, sex, insulin use	1.38 (0.86–2.24)	0.179	1.10 (0.67–1.79)	0.695	1.45 (0.87–2.44)	0.151	0.95 (0.59–1.52)	0.833
PC, age, sex, heart disease	1.73 (1.10–2.73)	0.017	1.14 (0.89–2.23)	0.133	1.66 (1.01–2.72)	0.043	1.15 (0.74–1.79)	0.529
PC, age, sex, nephropathy	1.85 (1.17–2.91)	0.008	1.50 (0.95–2.38)	0.080	1.77 (1.08–2.89)	0.023	1.19 (0.76–1.85)	0.428
PC, age, sex, DM ≥ 20 years	1.78 (1.13–2.79)	0.012	1.43 (0.90–2.52)	0.124	1.73 (1.06–2.84)	0.027	1.18 (0.76–1.84)	0.449

PC: poor glycemic control; DM: diabetes mellitus; CI: confidence interval; METs: metabolic equivalents; OR: odds ratio.

## Data Availability

The data sets used to support the findings of this study are available from the corresponding author upon reasonable request.

## Appendix A

Table A1 shows the Chi-square statistics and the p-value of the Hosmer–Lemeshow goodness-of-fit test of the regression models in Table 1. The results show that all models had a good fit, except for some logistic models to the response variable of low gait speed. The models on which we base our conclusions had a good fit.

**Table A1:** Hosmer–Lemeshow test for goodness of fit for logistic regression models.

	Sarcopenia		Low Muscle Strength		Low Muscle Mass		Low Gait Speed	
	χ^2^ (df)	*p*-Value	χ^2^ (df)	*p*-Value	χ^2^ (df)	*p*-Value	χ^2^ (df)	*p*-Value
PC, age, sex	15.25 (8)	0.054	8.29 (8)	0.405	1.98 (8)	0.982	11.53 (8)	0.173
PC, age, sex, comorbidity	5.29 (8)	0.725	5.05 (8)	0.752	9.33 (8)	0.315	15.87 (8)	0.044
PC, age, sex, malnutrition	9.26 (8)	0.320	6.12 (8)	0.634	5.51 (8)	0.702	13.39 (8)	0.099
PC, age, sex, frailty	11.72 (8)	0.165	4.79 (8)	0.780	5.93 (8)	0.655	2.95 (8)	0.937
PC, age, sex, DM complication	12.11 (8)	0.146	6.82 (8)	0.555	4.88 (8)	0.770	17.79 (8)	0.023
PC, age, sex, activity ≤ 5 METs	4.86 (8)	0.772	3.50 (8)	0.899	2.68 (8)	0.953	3.92 (8)	0.915
PC, age, sex, insulin use	12.13 (8)	0.146	5.79 (8)	0.671	4.57 (8)	0.802	7.77 (8)	0.456
PC, age, sex, heart disease	10.04 (8)	0.262	8.94 (8)	0.348	3.83 (8)	0.872	18.18 (8)	0.020
PC, age, sex, nephropathy	7.66 (8)	0.467	8.65 (8)	0.373	13.1 (8)	0.108	11.8 (8)	0.168
PC, age, sex, DM ≥ 20 years	12.51 (8)	0.130	9.27 (8)	0.320	5.95 (8)	0.653	14.45 (8)	0.071

χ^2^: Chi-square statistics; df: degrees of freedom; PC: poor glycemic control; DM: diabetes mellitus; METs: metabolic equivalents.
